# Epistatic Role of the *MYH9/APOL1* Region on Familial Hematuria Genes

**DOI:** 10.1371/journal.pone.0057925

**Published:** 2013-03-14

**Authors:** Konstantinos Voskarides, Panayiota Demosthenous, Louiza Papazachariou, Maria Arsali, Yiannis Athanasiou, Michalis Zavros, Kostas Stylianou, Dimitris Xydakis, Eugenios Daphnis, Daniel P. Gale, Patrick H. Maxwell, Avraam Elia, Cristian Pattaro, Alkis Pierides, Constantinos Deltas

**Affiliations:** 1 Molecular Medicine Research Center and Laboratory of Molecular and Medical Genetics, Department of Biological Sciences, University of Cyprus, Nicosia, Cyprus; 2 Department of Nephrology, Nicosia General Hospital, Nicosia, Cyprus; 3 Department of Nephrology, University of Crete, Heraklion, Greece; 4 Centre for Nephrology, University College London, London, United Kingdom; 5 Division of Medicine, University College London, London, United Kingdom; 6 Department of Pediatrics, Archbishop Makarios III Hospital, Nicosia, Cyprus; 7 Institute of Genetic Medicine, European Academy Bozen/Bolzano (EURAC), Bolzano, Italy – Affiliated Institute of the University of Lubeck, Lubeck, Germany; 8 Department of Nephrology, Hippocrateon Hospital, Nicosia, Cyprus; Biomedical Research Foundation of the Academy of Athens, Greece

## Abstract

Familial hematuria (FH) is explained by at least four different genes (see below). About 50% of patients develop late proteinuria and chronic kidney disease (CKD). We hypothesized that *MYH9/APOL1*, two closely linked genes associated with CKD, may be associated with adverse progression in FH. Our study included 102 thin basement membrane nephropathy (TBMN) patients with three known *COL4A3/COL4A4* mutations (cohort A), 83 CFHR5/C3 glomerulopathy patients (cohort B) with a single *CFHR5* mutation and 15 Alport syndrome patients (cohort C) with two known *COL4A5* mild mutations, who were categorized as “Mild” (controls) or “Severe” (cases), based on renal manifestations. E1 and S1 *MYH9* haplotypes and variant rs11089788 were analyzed for association with disease phenotype. Evidence for association with “Severe” progression in CFHR5 nephropathy was found with *MYH9* variant rs11089788 and was confirmed in an independent FH cohort, D (cumulative p value = 0.001, odds ratio = 3.06, recessive model). No association was found with *APOL1* gene. Quantitative Real time PCR did not reveal any functional significance for the rs11089788 risk allele. Our results derive additional evidence supporting previous reports according to which *MYH9* is an important gene *per se*, predisposing to CKD, suggesting its usefulness as a prognostic marker for young hematuric patients.

## Introduction

The differential diagnosis in 2012 for familial hematuria (FH) includes mostly the *COL4A3/A4* heterozygous carriers that exhibit thin basement membrane nephropathy (TBMN) with lifelong microscopic hematuria [1]–[5] and a newly described disease, CFHR5 nephropathy, with isolated C3 mesangial deposits [6], [7]. Other still unknown causes may exist.

Heterozygous mutations in the *COL4A3/COL4A4* genes are said to account for up to 40–50% of families with TBMN [1]–[5]. CFHR5 nephropathy is also an autosomal dominant disease with an up to now unknown prevalence, although it is highly prevalent in Cyprus [6], [7]. We and others demonstrated that more than 50% of these patients develop added proteinuria and chronic kidney disease (CKD) after the age of 30 and with a broad phenotypic spectrum [8]–[10]. Interestingly, X-linked Alport syndrome cases (male patients) with mild mutations in the *COL4A5* gene, often present as phenocopies of TBMN, with a wide spectrum of phenotypes [11]–[17]. We hypothesize that this great phenotypic heterogeneity is largely due to modifier genes [10].

Studies in animal models for TBMN and Alport syndrome support the existence of genetic loci that influence disease progression [18], [19]. In humans, studies by Tonna et al (2008) and Voskarides et al (2012) provide evidence that *NPHS2*-R229Q predisposes to proteinuria in TBMN [20], [21]. Additionally, Papagregoriou et al (2012) investigated potential microRNAs' target sites in hematuric patients and found that a miR-1207-5p binding site variant abolishes regulation of *HBEGF* and is associated with disease severity in CFHR5 nephropathy [22].

In this study, we took advantage of the extended founder effects we have observed among the Greek-Cypriot population, to investigate whether genetic polymorphisms of the *MYH9/APOL1* region can act as modifiers for the FH phenotype. Genome-wide association studies (GWAS) have identified *MYH9* (Myosin Heavy Chain 9) and its closely linked gene *APOL1*, as major susceptibility genes predisposing towards end stage kidney disease (ESKD), in various types of renal diseases (idiopathic focal segmental glomerulosclerosis, HIV-associated nephropathy, hypertensive nephrosclerosis, diabetic nephropathy, IgA nephropathy progression), in African-Americans, European-Americans, Europeans, Hispanic-Americans and Chinese [23]–[27]. For the present work our cohorts included patients with a monogenic primary form of FH, TBMN, Alport Syndrome and CFHR5 nephropathy. Our results demonstrate a likely contribution of *MYH9* variant rs11089788 in the progression of CKD when co-inherited with CFHR5 nephropathy, but cast doubt on the recently alleged association with variation in the *APOL1* gene that is closely linked to *MYH9*.

## Materials and Methods

### Study Cohorts - Clinical Assessment and Study Outcomes

The study cohorts are presented in Table 1. Patients of all four cohorts were categorized as having “Mild” or “Severe” disease. Our main cohorts include 102 adult TBMN patients, 83 adult CFHR5 patients and 15 Alport syndrome patients. These cohorts are unique worldwide because they include a large number of patients with common mutations due to founder effects. Among 102 TBMN patients, 77 carry mutation *COL4A3*-p.G1334 and 19 carry mutation *COL4A3*-p.G871C. All 83 CFHR5 patients carry the same exon 2–3 duplication. This situation offers an advantage as regards reduced genomic background complexity based on common ethnicity, thereby facilitating the search for genetic modifiers in relatively smaller cohorts. Since young individuals with apparently mild disease could develop severe disease when older, we excluded young patients without evidence of severe disease (Table 1). Mild disease was characterized by the presence of only microscopic hematuria and up to low grade proteinuria, repeatedly under 300 mg/day and no CKD. “Severe” disease was characterized by hematuria plus proteinuria ≥500 mg/day, or hematuria plus proteinuria plus CKD, or ESKD (Table 1). CKD was defined as an elevated serum creatinine over 1.5 mg/dL. Patients with borderline proteinuria and another concomitant renal disease (*e.g.*, over five years of diabetes, vesicoureteral reflux etc), or severe patients at the extreme of body weight (outside ±2 SD of the cohort mean) were excluded. For Alport syndrome patients, disease severity is defined by most researchers according to age at ESKD, so the severe group included patients that reached ESKD below the age of 40 years. Cohort D was genotyped for replication purposes, including FH patients not genetically studied yet. The study was approved by the Cyprus National Bioethics Committee and participants gave their signed informed consent, unless they were included anonymously after testing for purely diagnostic purposes.

**Table 1:** Description of cohorts and patients under study.

Patient group (cohort)	Origin	N	Mild				Severe			
			N (%)	Age: mean (SD)	Females: N (%)	with ESKD: N (%)	N (%)	Age: mean (SD)	Females: N (%)	with ESKD: N (%)
**A.** Heterozygous mutation carriers of *COL4A3*-G1334E or *COL4A3*-G871C or *COL4A4*-3854delG^1^	Cyprus	102	44 (43%)	60.3 (±10.3)	26 (59%)	0	58 (57%)	62.6 (±12.9)	26 (44%)	20 (34%)
**B.** Heterozygous mutation carriers of *CFHR5* Exons 2–3 duplication^2^	Cyprus	83	48 (58%)	57.5 (±12.9)	29 (60%)	0	35 (42%)	58.4 (±11.1)	8 (23%)	20 (57%)
**C.** XLAS male patients, mutation carriers of *COL4A5*-P628L or *COL4A5*-G624D^3^	Cyprus, Greece	15	11 (41%)	50.8 (±5.3)	0	5 (45%)	4 (59%)	50.8 (±5.3)	0	4 (100%)
**D.** Familial cases of MH^4^	Cyprus, Greece	67	33 (49%)	53.8 (±8.9)	26 (79%)	0	34 (51%)	56.4 (±12.8)	13 (38%)	11 (32%)

Please note that cohort B1 is the male only patients with CFHR5 nephropathy.

MH: Microscopic Hematuria, ESKD: End Stage Kidney Disease, XLAS: X-linked Alport syndrome.

1 “Mild” patients born before 01/1963. Gender difference (Mild vs Severe) is not significant (p = 0.141).

2 “Mild” patients born before 01/1975. Gender difference (Mild vs Severe) is significant (p = 0.001).

3 “Severe” patients: ESKD≤40 yo.

4 “Mild” patients born before 01/1979. Gender difference (Mild vs Severe) is significant (p = 0.001).

### Single Nucleotide Polymorphisms (SNPs) genotyping – Haplotype analysis

We genotyped *MYH9* SNPs that were previously shown to be strongly associated with renal failure in Caucasian populations (Table 2; it includes information on the PCR-RFLP genotyping assays): two lying on the E1 risk haplotype (rs4821481 and rs4821480), one lying on the S1 risk haplotype (rs2413396) and the rs11089788 variant that is found in the 5′-end of *MYH9* (IVS3 -5801C/A) – identified by a meta-analysis study – associated with serum creatinine levels in three Caucasian populations (Table 2).

**Table 2:** Information about the *MYH9* non-coding SNPs and the non-synonymous *APOL1* SNPs, genotyped in this study.

SNP	Risk haplotype	Forward primer	Reverse primer	Tm (°C)	Restriction enzyme	Cleavage products (bp)	Known associations in Caucasian populations
*MYH9* rs4821481 (T/C)	E1	GAAGAGTACCCCGTATCTCAACAC	AGCATGAGGGCTTCTGCTTAACT	65	*BfaI*	132+115 (T allele)	1. Non-diabetic ESKD in Hispanic Americans [26] 2. Diabetic nephropathy in European Americans [33]
*MYH9* rs4821480 (T/G)	E1	CTCATGCTTGTTCTTGAGCTTG	AGAACAGAAAGCAAGGAGAGCAG	66	*DraI*	261+291 (T allele)	1. Non-diabetic ESKD in Hispanic Americans [26] 2. Diabetic nephropathy in European Americans [33] 3. Non-diabetic CKD in European Americans [33]
*MYH9* rs2413396 * (T/C)	S1	CCAGCACCTCCCCGTGA	GTGGAGAAGGTGATGCAGGAG	65	*HaeII*	109+21 (C allele)	1. Non-diabetic ESKD in Hispanic Americans [33]
*MYH9* rs11089788 (C/A)	No strong LD (<0.3) with any neighboring SNPs, according to HapMap data	GATGTCCCATCCAATTGTTTTC	GATTATGGCCTAAAAAGGCAACTG	59	*BslI*	230+158 (C allele)	1. Association with serum creatinine levels in three Caucasian populations [33]
*APOL1* E149K	-	CTGGTTTCTGAAAGAGTTTCCTCGGTTGAAAA**T**T **	GCTGTGATTCCCAACTCCATCCCAGGTTCCAA	60	*MseI*	207+33 (K allele)	-
*APOL1* M227I	-	CGTATTAGTCAGGATGGTCTCAATC	AAAGTTGGATATGTTCTCACCCAAA	65	*BtgI*	505+115 (M allele)	-
*APOL1* R254K	-	CTGGTCATCAAAAGCCTTGACAAATTGAAGGAGGT**T**A **	ATTAACCCTCTCCACCTGTTCACCGCTTTCAGCTG	67	*MseI*	201+35+4 (K allele)	-

* Genotyping of rs2413396 was performed according to [27].

** Modified nucleotides for creating restriction sites are in bold and underlined.

DNA samples from 15 patients (six from cohort A, five from cohort B and four from cohort C), representing all three rs11089788 genotypes, were selected for PCR amplification (primers available on request) and DNA re-sequencing (in ABI PRISM™ 3130) of the last exon of *APOL1*, where most of non-synonymous SNPs are located, according to electronic databases (www.ensemble.org). Three of the five non-synonymous SNPs detected, were genotyped in cohorts A, B and D. Haplotype analysis was performed by Haploview software (http://www.broad.mit.edu/mpg/haploview/).

### Statistical analysis

Genotypic statistical analysis and odds ratios calculations were performed by SPSS v.13 (IBM, USA). Both dominant and recessive models of inheritance were tested for significance where this was possible. P values were calculated by Pearson Chi-Square test. Fisher's Exact Test (2-sided) was used where genotype values less than 10 existed. The significance level, alpha, was set to 0.05. We decided to genotype the entire cohort B (it includes males and females with CFHR5 nephropathy), only for SNPs that would give a p value close to 0.1 for cohort B1 (B1 are only the males with CFHR5 nephropathy; mostly males proceed to CKD in this type of FH).

### Real Time PCR

mRNA was isolated from peripheral blood leukocytes using the QIAamp RNA Blood Mini Kit (Qiagen, Germany) from: three male healthy volunteers genotyped as CC for rs11089788, three male healthy volunteers genotyped as AA for rs11089788 and three male “severe” hematuric patients genotyped as CC for rs11089788. RNA integrity and concentration were assessed by gel electrophoresis and spectrophotometrically (Nanodrop technologies), respectively. 200 ng of total RNA from each sample was reverse transcribed (ProtoScript™ by New England Biolabs, UK). The quantitative Real-Time PCR (qRT-PCR) amplifications were performed in duplicate on the LightCycler® system (Roche Diagnostics and Applied Sciences) using SYBR Green (Applied Biosystems, California, USA) in a reaction volume of 20 µl and primers according to Liang et al (2011) [28]. Relative quantification analysis was carried out on the LightCycler® Software 4.1. The endogenous reference gene *GAPDH* was used for normalisation of the results. Statistical significance was checked by independent t-test, through the SPSS v.13 package (IBM, USA).

## Results

### CC genotype of *MYH9* rs11089788 confers significant risk for proteinuria and CKD

None of the analyzed *MYH9* SNPs was significantly associated with phenotypes in any of our initial cohorts. In accordance with previous publications, *MYH9* rs4821480 was found to be in complete linkage disequilibrium (LD) with *MYH9* rs4821481, after genotyping 85 samples of cohort A; thus it was not genotyped any further. *MYH9* rs2413396 was found in partial LD with *MYH9* rs4821481 (see frequencies in Table 3, LD value was not calculated). *MYH9* rs11089788 gave a p value close to 0.1 in cohort B1 (Table 4). Hence, cohort B (B = B1 plus the female patients, see Methods) and D were analyzed only for *MYH9* rs11089788. For cohort B, *MYH9* rs11089788 gave a p-value of 0.015 (OR = 3.18) under a recessive model. Cohort D replicated the significance for *MYH9* rs11089788 (p = 0.024) under a recessive model of inheritance (Table 5). The significance was highly increased for the sum of cohorts B+D for *MYH9* rs11089788 (p = 0.001 and OR = 3.06, see Table 5).

**Table 3:** Genotype distribution of the studied *MYH9* variants, by cohort and by severity.

SNP	*MYH9* rs4821481			*MYH9* rs2413396			*MYH9* rs11089788			
Mild	TT	TC	CC	TT	TC	CC	AA	AC	CC	n
Cohort A	28 (64%)	14 (32%)	2 (4%)	29 (66%)	14 (32%)	1 (2%)	14 (32%)	21 (48%)	9 (20%)	44
Cohort B1	17 (89%)	2 (11%)	0 (0%)	16 (84%)	3 (16%)	0 (0%)	6 (32%)	9 (47%)	4 (21%)	19
Cohort B	-	-	-	-	-	-	**15 (31%)**	**22 (46%)**	**11 (23%)**	48
Cohort C	8 (73%)	3 (27%)	0 (0%)	8 (73%)	3 (27%)	0 (0%)	3 (27%)	3 (27%)	5 (46%)	11

Severe	TT	TC	CC	TT	TC	CC	AA	AC	CC	
Cohort A	40 (69%)	18 (31%)	0 (0%)	37 (64%)	19 (33%)	2 (3%)	15 (26%)	28 (48%)	15 (26%)	58
Cohort B1	24 (89%)	3 (11%)	0 (0%)	21 (78%)	6 (22%)	0 (0%)	3 (11%)	12 (44.5%)	12 (44.5%)	27
Cohort B	-	-	-	-	-	-	**3 (8.5%)**	**15 (43%)**	**17 (48.5%)**	35
Cohort C	4 (100%)	0 (0%)	0 (0%)	4 (100%)	0 (0%)	0 (0%)	3 (75%)	0 (0%)	1 (25%)	4

**Table 4:** Genotype associations for the three *MYH9* variants genotyped in this study.

Variant	p value	OR (95% CI)	p-value	OR (95% CI)
***MYH9*** ** rs4821481**	TT+TC vs CC		TT vs TC+CC	
Patients cohort A	0.184*	**	0.572	0.79 (0.34, 1.80)
Patients cohort B1	-	-	1.000*	1.06 (0.16, 7.06)
Patients cohort C	-	-	0.516*	**
Sum (A+B1+C)	0.205*	**	0.487	0.78 (0.39, 1.58)
***MYH9*** ** rs2413396**	TT+TC vs CC		TT vs TC+CC	
Patients cohort A	1.000*	1.54 (0.14, 17.50)	0.825	1.10 (0.48, 2.50)
Patients cohort B1	-	-	0.716*	1.52 (0.33, 7.04)
Patients cohort C	-	-	0.516*	**
Sum (A+B1+C)	1.000*	1.68 (0.15, 18.88)	0.785	1.10 (0.56, 2.17)
***MYH9*** ** rs11089788**	AA+AC vs CC		AA vs AC+CC	
Patients cohort A	0.639*	1.36 (0.53, 3.47)	0.509	1.34 (0.56, 3.18)
Patients cohort B1	0.126*	3.00 (0.79, 11.45)	0.133*	3.69 (0.79, 17.25)
Patients cohort B	**0.015**	**3.18 (1.24, 8.17)**	**0.016** *	**4.85 (1.28, 18.36)**
Patients cohort C	0.604*	0.40 (0.01, 5.15)	0.235*	0.125 (0.01, 1.72)
Sum (A+B+C)	0.129	1.61 (0.87, 2.98)	0.131	1.63 (0.86, 3.09)

P-values were calculated by Pearson Chi-Square test. The whole CFHR5 cohort (B) was genotyped only for *MYH9* rs11089788, the only SNP that gave p-value close to 0.1 for the male CFHR5 patients (B1).

* P-values calculated by Fisher's Exact Test (2-sided) due to existence of genotypes values less than 10.

** Odds ratio (OR) cannot be estimated due to zero genotypic values in the “Severe” category.

**Table 5:** *MYH9* rs11089788 statistical analysis for a replicate cohort (D) and for the sum of cohorts B and D, that gave statistical significance.

		*MYH9* rs11089788			P value, Odds ratio	P value, Odds ratio
Cohort	n	AA	AC	CC	AA+AC vs CC	AA vs AC+CC
D – “Mild”	33	7 (21%)	18 (55%)	8 (24%)	P = 0.024, OR = 3.52 (1.24, 9.97)	P = 0.765, OR = 1.26 (0.37, 4.23)
D – “Severe”	34	6 (18%)	10 (29%)	18 (53%)		
B+D – “Mild”	83	23 (28%)	40 (48%)	**20 (24%)**	**P = 0.001, OR = 3.06** (1.54, 6.10)	P = 0.049, OR = 2.26 (0.99, 5.16)
B+D – “Severe”	69	10 (15%)	25 (36%)	**34 (49%)**		

Patients in cohort D belong to families that segregate microscopic hematuria but no known mutation has been found in any of the genes *COL4A3*, *COL4A4*, or *CFHR5*, so far.

### Non-synonymous SNPs found on the last exon of *APOL1*

**Figure 1:**
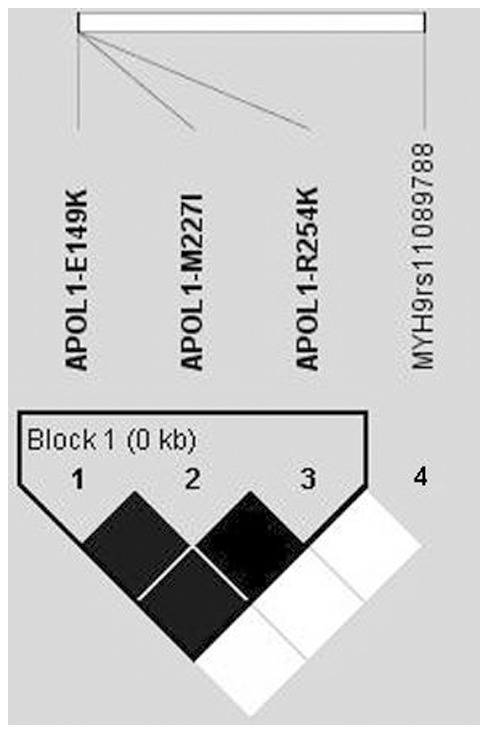
Linkage disequilibrium plots show that E149K, M227I and R254K in *APOL1* gene are in complete linkage disequilibrium (D′ = 1), but are not linked with *MYH9* rs11089788.

Re-sequencing of the last *APOL1* exon revealed five non-synonymous SNPs: p.E149K (rs2239785), p.M227I (rs136175), p.R254K (rs136176), p.G270D (rs73403889) and p.D336N (rs16996616). Those SNPs are not among the published ones to be associated with CKD [29], [30]. *APOL1* p.E149K, p.M227I and p.R254K [all reported with significant frequency only in Caucasian samples (http://hapmap.ncbi.nlm.nih.gov/)] were genotyped for the cohorts A, B and D. No significant association was observed (results not shown). All three *APOL1* SNPs were in complete LD, constructing a haplotype block (results compatible with HapMap data), but they were not linked with rs11089788 (Figure 1).

### Expression of *MYH9* in rs11089788 “CC” and “AA” patients

No statistical significance was observed when comparing the means of the blood mRNA values among the genotypic groups, (see Methods, results not shown) despite that there was a trend for an increase of the CCs over the AAs.

## Discussion

Genotype CC of rs11089788, located on the 5′-end of *MYH9*, was found to be significantly associated with bad progression in FH in two different cohorts. No significant association was observed for SNPs representing E1 και S1 haplotypes. This may be expected as these haplotypes were mainly associated with renal failure in populations out of Europe [23], [24]. On the other hand, *MYH9* rs11089788 was previously associated with high serum creatinine levels in Europeans [25] and with progression of IgA nephropathy in Chinese [27]. CFHR5 nephropathy and IgA nephropathy possibly share common pathophysiology features due to their immune origin. Pending confirmation with other studies, our study suggests that rs11089788 is a negative prognostic factor in CFHR5 nephropathy and possibly in other glomerular hematuric diseases. This result was confirmed in a second cohort (D), including hematuric patients of various etiologies. Considering the two cohorts together, the results show that a hematuric patient with the CC genotype (recessive model) has a 3.06-fold increased likelihood (p = 0.001) for proteinuria and renal failure (Table 5). This enables us to speculate that certain FH types may share common risk factors as regards disease progression.

It is useful to underline that for certain types of FH, gender is an important cofounder in the disease progression in association with the modifier genes. In this study, difference in gender percentages is significant for cohort B (CFHR5 patients) and cohort D. In a previous publication we discuss in detail the high risk of male CFHR5 patients for progression to renal failure, comparing also the survival rates of renal function in the two genders [6], [17]. All in all however, in accordance with the previous literature, the significance we describe here is still valid when taking into consideration the gender.

Recent studies give evidence that three coding SNPs on the last exon of *APOL1* gene, lying on extended haplotypes with the *MYH9* SNPs (haplotypes E1, F1 and S1), may be the actual cause for predisposition to renal failure. On the other hand, O'Seaghdha et al (2011) found association of rs4821480 with CKD in European-Americans [31], but did not find an association with any of the *APOL1* SNPs reported by Genovese et al (2010) and Tzur et al (2010), suggesting that variation in *APOL1* may not be the complete explanation [29], [30]. We screened the last exon of *APOL1*, but the identified SNPs were not in LD with either of the two alleles of rs11089788, despite a strong LD existing between them (Fig. 1), this being compatible with results of O'Seaghdha et al (2011) [31]. Additionally, Freedman et al (2011) found that homozygotes for *MYH9* E1 haplotype are protected from Diabetic Nephropathy when predisposed by *FRMD3* SNPs; this is in contrast to the non-carriers of *MYH9* E1 that are prone to Diabetic Nephropathy when carrying the *FRMD3* SNPs. These data are indicative of more complex and unpredictable gene interactions and are supportive of our results, signifying the genetic modifying influence of *MYH9* SNPs. Non-coding SNPs on *MYH9* may have an unknown effect on *MYH9* e.g. in its expression, even though we did not find such an effect by a quantitative real time PCR approach. Some of those SNPs may affect proper splicing of *MYH9*, as Nelson et al (2010) found recently for rs2413396 and rs4821480 [32].

Our finding for rs11089788 may prove useful in FH cases, with early and effective interventions at early life, before symptoms worsen. However, it remains unclear how certain non-coding *MYH9* variants exert their epistatic role and are associated with renal failure. It does not escape our attention that despite the unique character of our cohorts as regards the reduced genetic complexity owing to the extensive founder mutations, the overall size of our patient cohorts is somewhat small, and unfortunately there is no easy way to remedy this inherent limitation of the population we serve. Future collaboration of multiple research centers will be necessary to increase the statistical power and replicate or annul our results.
